# Validity and reliability of a new tool to evaluate handwriting difficulties in Parkinson’s disease

**DOI:** 10.1371/journal.pone.0173157

**Published:** 2017-03-02

**Authors:** Evelien Nackaerts, Elke Heremans, Bouwien C. M. Smits-Engelsman, Sanne Broeder, Wim Vandenberghe, Bruno Bergmans, Alice Nieuwboer

**Affiliations:** 1 Department of Rehabilitation Sciences, KU Leuven, Leuven, Belgium; 2 Department of Health and Rehabilitation Sciences, University of Cape Town, Cape Town, South Africa; 3 Department of Neurosciences, KU Leuven, Leuven, Belgium; 4 Department of Neurology, University Hospitals Leuven, Leuven, Belgium; 5 Department of Neurology, A.Z. Sint-Jan Brugge-Oostende, Bruges, Belgium; Tokai University, JAPAN

## Abstract

**Background:**

Handwriting in Parkinson’s disease (PD) features specific abnormalities which are difficult to assess in clinical practice since no specific tool for evaluation of spontaneous movement is currently available.

**Objective:**

This study aims to validate the ‘Systematic Screening of Handwriting Difficulties’ (SOS-test) in patients with PD.

**Methods:**

Handwriting performance of 87 patients and 26 healthy age-matched controls was examined using the SOS-test. Sixty-seven patients were tested a second time within a period of one month. Participants were asked to copy as much as possible of a text within 5 minutes with the instruction to write as neatly and quickly as in daily life. Writing speed (letters in 5 minutes), size (mm) and quality of handwriting were compared. Correlation analysis was performed between SOS outcomes and other fine motor skill measurements and disease characteristics. Intrarater, interrater and test-retest reliability were assessed using the intraclass correlation coefficient (ICC) and Spearman correlation coefficient.

**Results:**

Patients with PD had a smaller (p = 0.043) and slower (p<0.001) handwriting and showed worse writing quality (p = 0.031) compared to controls. The outcomes of the SOS-test significantly correlated with fine motor skill performance and disease duration and severity. Furthermore, the test showed excellent intrarater, interrater and test-retest reliability (ICC > 0.769 for both groups).

**Conclusion:**

The SOS-test is a short and effective tool to detect handwriting problems in PD with excellent reliability. It can therefore be recommended as a clinical instrument for standardized screening of handwriting deficits in PD.

## Introduction

Writing problems often manifest themselves as the first symptom of Parkinson’s disease (PD). Although generally known as micrographia, i.e. ‘*an impairment of a fine motor skill manifesting mainly as a progressive or stable reduction in amplitude during a writing task* ‘[1], writing problems in PD can be more complex. They often include timing deficits, irregularities and breakdown of movement, as well as loss of amplitude [2–5]. Furthermore, different patients display varying degrees of variability in timing and/or amplitude, e.g. presenting as either a global reduction (consistent) or a gradual reduction in writing size (progressive) [6]. Therefore, a shift in terminology from micrographia to dysgraphia was recently suggested by Lettaneux *et al*. [4]. As handwriting is an important daily activity, writing problems constitute an important impairment of manual dexterity in daily life [1, 7].

A valid screening instrument that can assess and monitor these different components and can detect changes of performance in a clinical setting is currently lacking. To assess tremor, bradykinesia and rigidity, spiral drawings or tracking of pre-drawn figures have been used so far [8–10]. As these tasks rely on the continuous provision of feedback and are novel rather than automated, they are unlikely to reflect true writing [11]. Furthermore, these tasks do not include the more complex nature of the handwriting process, which incorporates both automated and controlled processes [12]. Recently, Smits *et al*. used a digitizer pen to record pen tip trajectories to assess movement time, velocity and size, as well as rest tremor during repeated character 'elelelel' and sentence writing [13]. As this approach required a specific digitizer pen, a toolkit was developed to assess static signatures of patients with PD [14]. However, due to the nature of a signature, usually comprising of one word, it is not possible to determine whether writing deficits are progressive. Although technology can provide detailed quantitative analyses of handwriting, not all clinicians will have access to it and the use of technology will often require an investment.

To accommodate the problems with existing evaluation methods for handwriting in PD, the validity of the ‘Systematic Screening of Handwriting Difficulties (SOS)’ was studied. This screening tool was originally developed to detect early writing problems in children and was composed of the six most sensitive items of the “Beknopte beoordelingsmethode voor kinderhandschriften (BHK)” [15]. It not only evaluates writing amplitude and velocity, but also additional writing parameters contributing to the quality of handwriting. The SOS-test is an easy-to-use test and only requires a blank page, pen and timer, making it feasible for use in daily practice. In this study, we aimed to investigate the construct validity of the SOS-test and whether it can discriminate between patients with PD and healthy controls. Secondly, we examined whether the SOS-test is a reliable tool by means of intrarater, interrater and test-retest analyses.

## Materials and methods

### Participants

In this cross-sectional study 87 patients with PD and 26 healthy controls were tested. Inclusion criteria for patients were: (i) diagnosis of PD according to the ‘UK Brain Bank Criteria’; (ii) Hoehn and Yahr (H&Y) stage I-IV in the on-phase of the medication cycle [16]; (iii) MiniMental State Examination (MMSE) > 24 [17]; and (iv) stable medication regimen. Participants were excluded in case of (i) interfering medical upper limb problems, such as arthritis or recent fractures of the hand; and (ii) the presence of a neurological disorder other than PD.

The study design and protocol were approved by the local Ethics Committee of the University Hospitals Leuven in accordance with The Code of Ethics of the World Medical Association (Declaration of Helsinki). After a full explanation of the procedure and prior to testing, written informed consent was obtained from all participants.

### Procedure

All participants performed the Dutch version of the SOS-test to assess daily life writing (also available in English and German) [15]. To assess construct validity, other fine motor skills were evaluated using the Manual Ability Measure (MAM-16) questionnaire[18] and Purdue Pegboard test [19]. In addition, the Edinburgh Handedness Inventory was completed [20]. Disease specific characteristics were evaluated using the Movement Disorder Society Unified Parkinson’s Disease Rating Scale part III (MDS-UPDRS-III) [21], and the H&Y staging scale. Sixty-seven of the 87 PD patients performed the SOS-test a second time, at least one week and at the most one month after the first test to determine test-retest reliability. Patients were tested during the on-phase of the medication cycle, i.e. approximately one hour after last medication intake. We chose to test patients during the on-phase, as this closely resembles the clinical practice. Patients were tested either at home or at the faculty of Rehabilitation Sciences of KU Leuven, a situation which was kept constant for each participant. Also, patients were tested at the same time of day on both test occasions and medication regimes did not change between tests.

### Systematic screening of handwriting difficulties

For the SOS-test, all participants received a printed text, along with a blank piece of paper to copy the text. Participants used a normal pen and the use of erasers was not allowed. During five minutes, participants had to copy as much as possible of the given text with the instruction to write as quickly and neatly as in daily life. Two different standard texts were used to avoid learning effects for the test-retest evaluation.

**Fig 1:**
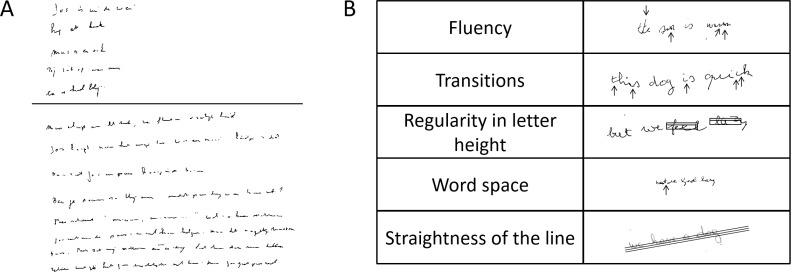
The SOS-test. (A) Example of a Dutch SOS-test of a patient with PD; (B) The subitems of the quality score in English. The arrows and horizontal lines indicate where problems were detected.

Mean writing size, writing speed and the quality of handwriting were determined. Writing speed was obtained by counting all letters written within five minutes, including letters that were crossed out. The first five lines, each containing one sentence, were used to evaluate the quality of handwriting, based on five criteria (**Fig 1**): (i) fluency in letter formation; (ii) connections between letters; (iii) regularity of letter size; (iv) space between words; and (v) straightness of the sentence. The total SOS-score was the sum of the scores on the five criteria, with higher scores reflecting worse quality of handwriting. Each criterion received a score ranging from zero to two. A score of zero was given when the handwriting problem did not occur or was only present in one sentence. A score of one was given if the problem appeared in two or three sentences and a score of two when the problem occurred in more than three sentences. Contrary to the scoring of the test for children, mean handwriting size was not incorporated into the total SOS-score as for PD the assumption that smaller handwriting reflects better performance is not applicable. The mean handwriting size (mm) was assessed separately, using the standardized template based on the first five lines.

### Statistical analyses

Two raters (EN and EH), blinded to the other rater’s results and disease status of the subjects, scored 180 copies (87 patients, 26 controls and 67 patients at re-test) of the SOS-test twice. Intrarater reliability was determined for both raters by comparing the first and second scoring. Interrater reliability was determined using the first scoring of both raters. Test-retest reliability was assessed for 67 patients with PD and was based on the first scoring of test one and two by the first rater (EN).

Normality of the data was assessed by means of QQ-plots, and histograms. Depending on the distribution, general characteristics of patients with PD and healthy controls were compared using an independent t-test or the non-parametric Mann-Withney-U test. For comparison of gender the Chi-squared test was used. Main outcomes of the SOS-test, i.e. score, size and speed, were compared between groups using a one-way ANCOVA with gender as a covariate, as this differed significantly between groups and has been shown to influence handwriting [22]. The assumption of normality was assessed by means of Q-Q plots, while the Levene’s test was used to determine equality of variances. Differences between groups for the subitems of the SOS-score, i.e. fluency, connections, regularity, space and straightness, were assessed by means of a Chi-squared test. Additionally, an exploratory subgroup analysis was done comparing patients with corresponding side of disease onset and writing hand (= congruent, N = 59) to patients with differing side of onset and writing hand (= incongruent, N = 28) for the main SOS-outcome variables.

Construct validity was assessed by correlating the main SOS outcomes (score, size and speed) with other measures for fine motor skills shown to differ between patients with PD and healthy controls, i.e. Purdue Pegboard and MAM-16 [23, 24], using Spearman correlation analysis for patients and controls separately. To investigate the possibility that cognition is reflected in the writing process, the main SOS outcomes were correlated with the MMSE scores. False Discovery Rate (FDR) corrections were applied to correct for multiple testing. In addition, for patients a Spearman correlation analysis was performed with clinical characteristics, i.e. disease duration, H&Y stage, total MDS-UPDRS-III and upper limb items of the MDS-UPDRS-III. For the latter score items 3.4, 3.5, 3.6, 3.15 and 3.16 were used. Additionally, an exploratory analysis was performed in which a distinction was made between sequential upper limb items (3.4–3.6) and tremor-related upper limb items (3.15–3.16). FDR-corrections were applied to correct for multiple testing.

Paired t-tests and Wilcoxon signed rank tests were used to look for systematic differences within and between raters and between test moments. Intrarater, interrater and test-retest reliability were evaluated using the Intraclass Correlation Coefficient (ICC) for single measures in a two-way random effects model with absolute agreement for the total SOS-score, size and speed. For interpretation of the ICC the criteria from Shrout and Fleiss were used, with <0.40 as poor, 0.40–0.75 as fair to good and >0.75 as excellent reliability [25]. In case of normal distribution, the standard error of measurement ($\mathrm{SEM}={\mathrm{SD}}_{\mathrm{pooled}}\times \sqrt{\left(1-\mathrm{ICC}\right)}$) and minimal detectable change ($\mathrm{MDC}=\mathrm{SEM}\times 1.96\times \sqrt{2}$) were calculated. In case of abnormally distributed data, Spearman correlation coefficients were used to indicate reliability. Landis and Koch’s cutoffs were used for interpretation [26]. Bland Altman plots were constructed to visualize the mean difference between two raters or tests for SOS score, size and speed.

For the sub-items of the quality score, Wilcoxon signed rank tests were used to look for systematic differences and Cohen’s kappa coefficient to evaluate the intrarater, interrater, and test–retest reliability using the Landis and Koch benchmarks [26]. In addition, the percentage of agreement was calculated. SPSS (version 23) was used for all analyses. The significance level was set at p<0.05 for all tests.

## Results

### Subjects

Demographics and clinical characteristics of the participants are specified in **Table 1**. Groups did not differ for age, handedness and MMSE score. There was a significant difference between groups for gender and fine motor skills, as measured by the MAM-16 (p<0.001) and Purdue Pegboard test (all p<0.001). These indicated greater fine motor skill problems in PD compared to healthy controls.

**Table 1:** General Characteristics.

	PD (N = 87)	CT (N = 26)	p-value
**Age (years)**	66 (58, 71)	62 (54, 71)	0.587
**Gender (♂/♀)**	61 / 26	8 / 18	<0.001
**Edinburg Handedness Inventory (%)**	100 (88.9, 100)	100 (90, 100)	0.406
**MMSE (0–30)**	29 (28, 30)	29 (29, 30)	0.110
**MAM-16 (0–64)**	59 (55.5, 61)	64 (64, 64)	<0.001
**Purdue Dominant hand**	9.2 ± 2.7	13.0 ± 2.2	<0.001
**Purdue Non-dominant hand**	9.1 ± 2.2	12.5 ± 2.3	<0.001
**Purdue Both hands**	13.4 ± 4.4	20.5 ± 4.7	<0.001
**Purdue Combination**	15.7 ± 4.9	25.6 ± 7.1	<0.001
**Disease duration (years)**	6 (2, 10)	-	-
**H&Y (1–5)**	2 (2, 2)	-	-
**MDS-UPDRS-III (0–132)**	28.4 ± 14.0	-	-
**LED (mg/24h)**	484.8 ± 335.9	-	-

In case of normal distribution and equality of variances Mean ± standard deviation is presented, otherwise Median (first, third quartile) is displayed. **Abbreviations**: CT = healthy control; H&Y = Hoehn & Yahr stage; LED = Levodopa Equivalent Dose; MAM-16 = Manual Ability Measure; MDS-UPDRS-III = Movement Disorder Society Unified Parkinson’s Disease Rating Scale part III; MMSE = Mini Mental State Examination; PD = Parkinson’s disease.

### Group comparison and construct validity

**Fig 2:**
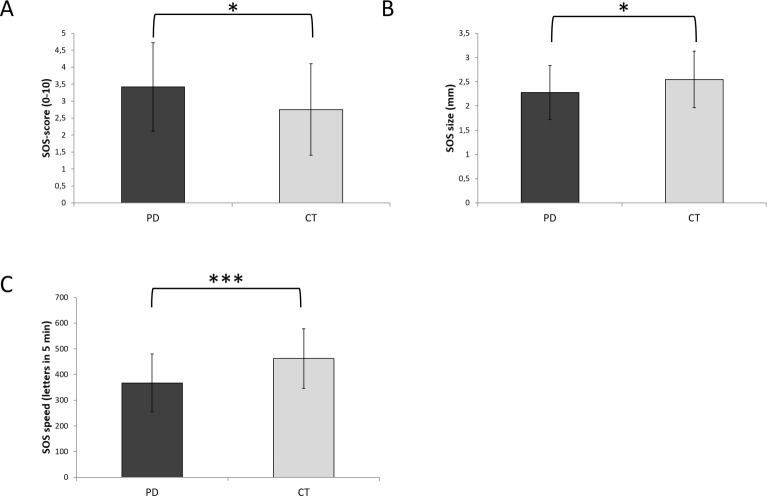
Difference between patients (PD) and healthy controls (CT). (A) SOS score; (B) size; (C) and speed. ** p < 0.01; *** p < 0.001.

When comparing PD patients and healthy controls, it was found that patients had a significantly higher SOS-score (F(1, 110) = 4.751; p = 0.031; *d* = 0.502), indicative of worse handwriting quality, as well as a smaller writing size (F(1, 110) = 4.205; p = 0.043; *d* = -0.475) and slower writing speed (F(1, 110) = 12.924; p < 0.001; *d* = -0.828) (**Fig 2**). Looking at the quality of handwriting into more detail, it was found that writing fluency (χ^2^ = 15.486; p<0.001) and regularity of letter height (χ^2^ = 16.098; p<0.001) were the items discriminating most between patients and controls with higher scores for patients. No significant differences were found for connections between letters, space between words or straightness of the sentence (all p>0.300).

The exploratory analysis, comparing patients depending on their side of onset, only revealed that the congruent group wrote more slowly compared to the incongruent group (t = -2.398; p = 0.019). No significant differences were found for handwriting quality or size.

Significant correlations, ranging from poor to moderate, showed that a higher SOS-score, smaller writing size and slower writing speed all correlated with worse fine motor skills, as measured by the Purdue Pegboard test, in patients with PD (**Table 2**). In healthy controls, slower writing speed also correlated with worse performance on the Purdue Pegboard test (**Table 2**). Additionally, a significant correlation was found between the MMSE score and writing speed in PD patients, with slower writing in more cognitively challenged patients. Furthermore, in patients a higher SOS-score correlated moderately with a longer disease duration, worse overall disease severity (MDS-UPDRS-III total score) and upper limb tremor severity (items 3.15–3.16 of the MDS-UPDRS-III) (**Table 2**). In addition, writing more slowly was moderately correlated with a worse disease severity (H&Y and MDS-UPDRS-III total score) and difficulties with sequential upper limb movements (items 3.4–3.6 of the MDS-UPDRS-III). Finally, writing size tended to decrease with increasing H&Y stage.

**Table 2:** Correlation analysis.

Parameter	SOS score		SOS speed		SOS size	
	PD	CT	PD	CT	PD	CT
	**Fine motor and cognitive skills**					
**MAM-16**	NS	NS	NS	NS	0.203(*)	NS
**Purdue**						
**Dominant hand**	NS	NS	0.420***	NS	0.200(*)	NS
**Non-dominant hand**	-0.265*	NS	0.291*	0.481(*)	NS	NS
**Both hands**	-0.374**	NS	0.324**	0.626*	NS	NS
**Combination**	-0.297*	NS	0.414***	0.569*	0.266*	NS
**MMSE**	NS	NS	0.425***	NS	NS	NS
	**PD specific**					
**Disease duration**	0.269*	-	NS	-	NS	-
**H&Y stage**	NS	-	-0.266*	-	-0.222(*)	-
**MDS-UPDRS-III**						
**Total**	0.276*	-	-0.376**	-	NS	-
**UL**	NS	-	-0.302*	-	NS	-
**UL sequential**	NS	-	-0.260*	-	NS	-
**UL tremor**	0.301*	-	NS	-	NS	-

**Abbreviations**: H&Y = Hoehn and Yahr stage; MAM-16 = Manual Ability Measure; MDS-UPDRS-III = Movement Disorder Society Unified Parkinson’s Disease Rating Scale part III; NS = not significant; PD = Parkinson’s disease; SOS = Systematic Screening of Handwriting Difficulties; UL = upper limb.

(*) p < 0.1

*p < 0.05

** p < 0.01

*** p < 0.001, FDR-corrected for multiple testing

### Reliability analysis

Bland and Altman plots are presented in **S1 Fig** for both interrater and test-retest analysis of the SOS-tests of patients with PD. No bias could be detected, as points distributed equally around zero for SOS-score, size and speed. For the SOS-score and size, overall scores fell within the limits of agreement. With regards to speed, two points were outside the limits of agreement in the interrater analysis. In addition, the limits of agreement were larger for test-retest analysis.

Interrater reliability was excellent for SOS-score and speed for both the patient and control group (**Table 3)**. Spearman correlation coefficients for writing size were also very good. More detailed analysis revealed that rater 1 gave a significantly lower SOS-score and larger size for both groups compared to rater 2 with small to medium effect sizes. No systematic difference was found for speed. Test-retest reliability was also excellent for SOS-score and speed with a very high Spearman correlation coefficient for writing size. No systematic differences were observed between sets of scores (**Table 3**). For intrarater reliability a comparable pattern could be observed for both raters, with excellent reliability for SOS-score, size and speed for both groups (**S1 Table**).

**Table 3:** Reliability analysis of SOS-test main outcome variables.

**Parameter SOS**	**Test/Rater 1 Mean (SD)**	**Test/Rater 2 Mean (SD)**	**t value**	**p-value**	**Effect size**	**ICC (95% CI)**	**SEM**	**MDC**
	**Interrater reliability**							
	**PD**							
**Speed**	367.7 ± 116.1	368.3 ± 115.8	1.536	0.127	-0.006	0.999 (0.999–1.000)	3.667	10.164
**Score**	3.5 ± 1.4	4.1 ± 1.9	5.292	0.000	-0.354	0.769 (0.633–0.848)	0.800	2.218
	**CT**							
**Score**	2.5 ± 1.4	3.5 ± 2.0	4.136	0.000	-0.783	0.783 (0.273–0.919)	0.805	2.231
	**Test–retest reliability**							
**Speed**	361.2 ± 119.2	368.0 ± 120.0	-1.268	0.209	0.057	0.965 (0.943–0.979)	22.374	62.018
**Score**	3.5 ± 1.3	3.5 ± 1.5	-0.108	0.915	0.007	0.806 (0.685–0.881)	0.618	1.712
	**Test/Rater 1 Median (IQR)**	**Test/Rater 2 Median (IQR)**	**Wilcoxon z value**	**p-value**	**Effect size**	**Spearman correlation coefficient**		
	**Interrater reliability**							
	**PD**							
**Size**	2.5 (2.0–2.5)	2.0 (2.0–2.5)	-4.897	0.000	-0.279	0.860		
	**CT**							
**Speed**	492.0 (362.3–533.0)	492.0 (363.0–533.0)	-0.431	0.666	-0.060	1.000		
**Size**	2.5 (2.5–3.0)	2.5 (2.0–3.0)	-1.848	0.065	-0.256	0.805		
	**Test–retest reliability**							
**Size**	2.5 (2.0–2.5)	2.0 (2.0–2.5)	-0.866	0.386	-0.075	0.807		

**Measurement units**: SOS score = unit less (0–10); SOS speed = letters written in 5 minutes; SOS size = mm. **Abbreviations**: CT = healthy control; ICC = Intraclass Correlation Coefficient; IQR = interquartile range; SD = standard deviation; SEM = Standard Error of Measurement; MDC = Minimal Detectable Change; SOS = Systematic Screening of Handwriting Difficulties; PD = Parkinson’s disease; 95% CI = 95% confidence interval. All ICC and Spearman correlation coefficients were significant at p < 0.001

Reliability of the sub-items of the quality score ranged from slight to almost perfect agreement, with agreement percentages ranging from 48–100% (**Table 4**). Detailed analysis showed a discrepancy between the Kappa statistic (0.115) and percentage agreement (91.558%) for the interrater reliability of the item of word space in PD. As 141 out of 154 SOS-tests received a score of zero from both raters, data distribution influenced the final Kappa statistic.

**Table 4:** Reliability analysis of subitems of the SOS quality score.

Parameter	Test/Rater 1 Median (IQR)	Test/Rater 2 Median (IQR)	Wilcoxon z value	p-value	Kappa (95% CI)	Agreement (%)
	**Interrater reliability**					
	**PD**					
**Fluency**	1.0 (1.0–2.0)	2.0 (1.0–2.0)	-3.584	0.000	0.209 (0.093–0.325)***	48.052
**Connections**	1.0 (1.0–2.0)	1.5 (1.0–2.0)	-3.194	0.001	0.438 (0.326–0.550)***	62.987
**Regularity**	1.0 (1.0–2.0)	1.0 (0.0–2.0)	-1.218	0.223	0.470 (0.366–0.574)***	63.636
**Word space**	0.0 (0.0–0.0)	0.0 (0.0–0.0)	-3.606	0.000	0.115 (-0.016–0.246)*	91.558
**Straightness**	0.0 (0.0–0.0)	0.0 (0.0–0.0)	-0.707	0.480	0.393 (0.219–0.567)***	79.221
	**CT**					
**Fluency**	0.0 (0.0–1.0)	1.0 (0.0–2.0)	-3.286	0.001	0.259 (0.047–0.471)*	50.000
**Connections**	1.0 (1.0–2.0)	2.0 (1.0–2.0)	-1.667	0.096	0.391 (0.099–0.683)**	65.385
**Regularity**	0.0 (0.0–1.0)	0.0 (0.0–1.0)	-1.134	0.257	0.508 (0.232–0.784)***	73.077
**Word space**	0.0 (0.0–0.0)	0.0 (0.0–0.0)	-1.000	0.317	1.000 (1.000–1.000)***	96.154
**Straightness**	0.0 (0.0–1.0)	0.0 (0.0–0.0)	-1.000	0.317	0.570 (0.202–0.938)**	84.615
	**Test–retest reliability**					
**Fluency**	1.0 (0.0–2.0)	1.0 (1.0–2.0)	-1.300	0.194	0.407 (0.231–0.583)***	61.194
**Connections**	1.0 (1.0–2.0)	1.0 (1.0–2.0)	-1.633	0.102	0.439 (0.255–0.623)***	64.179
**Regularity**	1.0 (1.0–1.0)	1.0 (1.0–1.0)	-0.536	0.592	0.407 (0.223–0.591)***	62.687
**Word space**	0.0 (0.0–0.0)	0.0 (0.0–0.0)	0.000	1.000	1.000 (1.000–1.000)***	100.000
**Straightness**	0.0 (0.0–0.0)	0.0 (0.0–0.0)	-0.302	0.763	0.448 (0.185–0.711)***	83.582

**Abbreviations**: CT = healthy control; IQR = interquartile range; PD = Parkinson’s disease; SOS = Systematic Screening of Handwriting Difficulties; 95% CI = 95% confidence interval.

* p < 0.05

** p < 0.01

*** p < 0.001

## Discussion

The present study was conducted to validate the SOS-test for PD and to evaluate whether it is a useful test to detect and monitor writing difficulties. An important innovative feature of this test is that it evaluates natural writing, addressing internally generated motor performance which is typically affected by PD. In addition, it only takes five minutes to complete and very little material is necessary, making it a useful standardized screening tool for clinical practice.

### Construct validity

Clear differences were observed between patients with PD and healthy controls with regards to SOS-score, size and speed, with patients performing worse on all three aspects, even though they were optimally medicated at the time of testing. Contrary to previously used evaluation methods [8–10, 14], the SOS-test evaluates prolonged writing, increasing the chance of capturing the automaticity deficit observed in PD [27]. Automatic movements have undergone a considerable amount of practice and are executed without attention directed towards the details of the movements. This type of habitual movement is known to highly rely on the functioning of the striatum, explaining the PD-specific difficulties as striatal dopamine depletion is a disease hallmark [28].

Analysis of the quality items revealed that fluency in writing and regularity of letter height were more affected in PD. The latter is in line with earlier findings, based on tablet technology, showing increased variability of writing size and decreasing letter height during writing in patients with PD while both in the on- and off-phase of the medication cycle [29–31]. Even though writing fluency in the current study was determined by means of sudden changes in movement directions, difficulties with writing fluency were shown previously in PD by means of an increased normalized jerk during writing-like movements [4, 32, 33]. Furthermore, it has to be noted that difficulties with fluency could be attributed to upper limb tremor, as 68 out of 87 patients presented with upper limb tremor. No significant differences were found for transitions between letters, the space between words or straightness of the sentences, which is in line with expectations, as these problems were also not reported earlier in PD raising the question whether these items may be redundant.

A correlation analysis was carried out to examine construct validity of the SOS-test. Weak to moderate correlations were found between the three main SOS outcome parameters and measures of manual dexterity. Writing is a complex motor skill that can be categorized as a form of manual dexterity, which is impaired in PD [24, 31]. However, the current results also show that writing performance has distinct components that are different from other fine motor skills. While writing is considered an automatically performed movement, the placing of pegs in the holes during the Purdue Pegboard test could be considered more goal-directed [28]. In addition, worse writing performance, specifically writing quality and speed, proved correlated with a longer disease duration and greater disease severity, confirming previous findings [1]. However, correlations in the current study were moderately high and there was merely a tendency towards a correlation between writing size and disease progression. One possible explanation is that even though patients from H&Y stage I-IV were included, 75.9% of patients were classified as H&Y stage II, while just 11.5% were in stage I, 11.5% in stage III and 1.1% in stage IV. As such, the more severe patients with PD were probably under-represented in this cohort. Interestingly, the SOS score correlated positively with the upper limb tremor-items of the MDS-UPDRS-III, while there was a negative correlation between SOS speed and the items on sequential upper limb movement. Both correlations, however, point towards a good construct validity of the SOS-test. A correlation between writing performance on the SOS-test and cognition was also investigated, as cognitive difficulties are common in PD, even in the early stages [34, 35]. We found that when patients experienced more cognitive difficulties, writing slowed down. Working memory plays an important role in the handwriting process [36]. As working memory capacity was shown to be reduced in PD, this could explain why patients with lower MMSE scores wrote more slowly [37, 38]. Although no longer significant after correction for multiple testing, healthy controls displayed a similar pattern, in line with cognitive impairments found as a result of healthy aging [39]. Overall, these findings point to the importance of intact cognition for writing speed and support the use of the SOS-test as a multi-component test of writing quality, rather than representing velocity alone.

Finally, recent work from our group has shown that differences between patients with and without freezing of gait can be detected with the SOS-test [29] and that the SOS-test is sensitive enough to detect improvements in writing size after intensive amplitude training [40]. These results suggest that the SOS-test can be used to monitor writing difficulties with time and detect intervention effects in PD.

### Reliability

Overall, results show an excellent intra- and interrater reliability for writing size and writing speed in PD and healthy controls. This can most likely be attributed to the objective criteria that are used for scoring, indicating that the SOS-test can be reliably used in both groups. Analysis with Bland-Altman plots showed that, in general, points were distributed equally around zero. Although the reliability of the overall quality score was excellent, the individual items should be interpreted with caution, as reliability varied from slight to almost perfect agreement.

Test-retest reliability in patients was excellent for the SOS-score, size and speed. Bland-Altman plots showed that the limits of agreement were larger for writing speed. One possible explanation is that this reflects the inherent variability of test performance due to fluctuations of symptoms, as was recently also suggested for the Instrumented Timed Up and Go Test [41].

### Study limitations and recommendations

The current study showed that the SOS-test can distinguish between patients with PD and healthy controls, however, the correlations with disease severity were less clear. Therefore, future research should include participants equally distributed over H&Y stages to investigate whether the SOS-test is sensitive enough to detect disease progression. In addition, handwriting has been suggested as a possible non-invasive biomarker for PD diagnosis [7]. Therefore, it would be interesting to include a newly-diagnosed *de novo* PD group in future studies to test whether the SOS-test is sensitive enough to detect early deficits. For this purpose, it would be necessary to assure consistency between side of onset and the hand with which the patient writes, as results revealed slower handwriting in patients who write with the hand that was initially affected. An alternative calculation of the SOS quality score may be needed to detect the differences between H&Y stages. For this purpose, we suggest to not only score whether or not problems occur in a sentence, but also to take into account the number of problems in each sentence. Scoring could also be made more sensitive by analyzing a larger portion of text. It has to be noted that writing speed on the SOS-test is calculated as the number of letters written in 5 min, while writing speed measured with specialized tablets is usually expressed in cm/s. Previous work has shown strong correlations between writing speed on the SOS-test and on a writing tablet [42, 43].

Secondly, recent research has suggested a partially different neural basis for consistent and progressive micrographia [44]. This suggests that a different rehabilitation approach might be necessary for either subtype. Further research is warranted to determine whether the SOS-test can be used to detect this difference.

Finally, future work is needed to uncover the possibilities of combining spontaneous writing with digitized tablets or pens [13, 42, 43]. Work is ongoing to validate these tools, but automation of calibration and analysis procedures need further refinement to allow clinical implementation (unpublished data). For now, we recommend to use the SOS-test in clinical practice with writing size as the main parameter for follow-up of PD patients due to the link between writing size and legibility of handwriting and the improvements found after intensive training. The quality score can provide additional information regarding fluency in letter formation and regularity of letter size. Future work should determine whether omitting the seemingly redundant items (word space and straightness of the sentence) would make the test more PD-specific without losing information, which would also aid future digitized versions. Finally, SOS writing speed is more informative about the degree of bradykinesia than handwriting legibility as such.

## Conclusion

We conclude that the SOS-test is a reliable tool with excellent construct validity, warranting its use as a clinical handwriting test in PD. Future work needs to be done to refine the test and make it even more specific for this population with the potential to serve as a diagnostic and progression biomarker.

## Supporting information

S1 Dataset (XLSX)

S1 Fig Bland-Altman plots for interrater and test-retest analysis. Panels A, B, D and E show less data points due to overlapping data.
(TIF)

S1 Table Reliability analysis of SOS-test main outcome variables. (DOCX)
