# Supplementary figures and images for: Validity and reliability of a new tool to evaluate handwriting difficulties in Parkinson’s disease
Source: PLoS One. 2017 Mar 2;12(3):e0173157. doi: 10.1371/journal.pone.0173157 (PMC5333892; doi:10.1371/journal.pone.0173157)

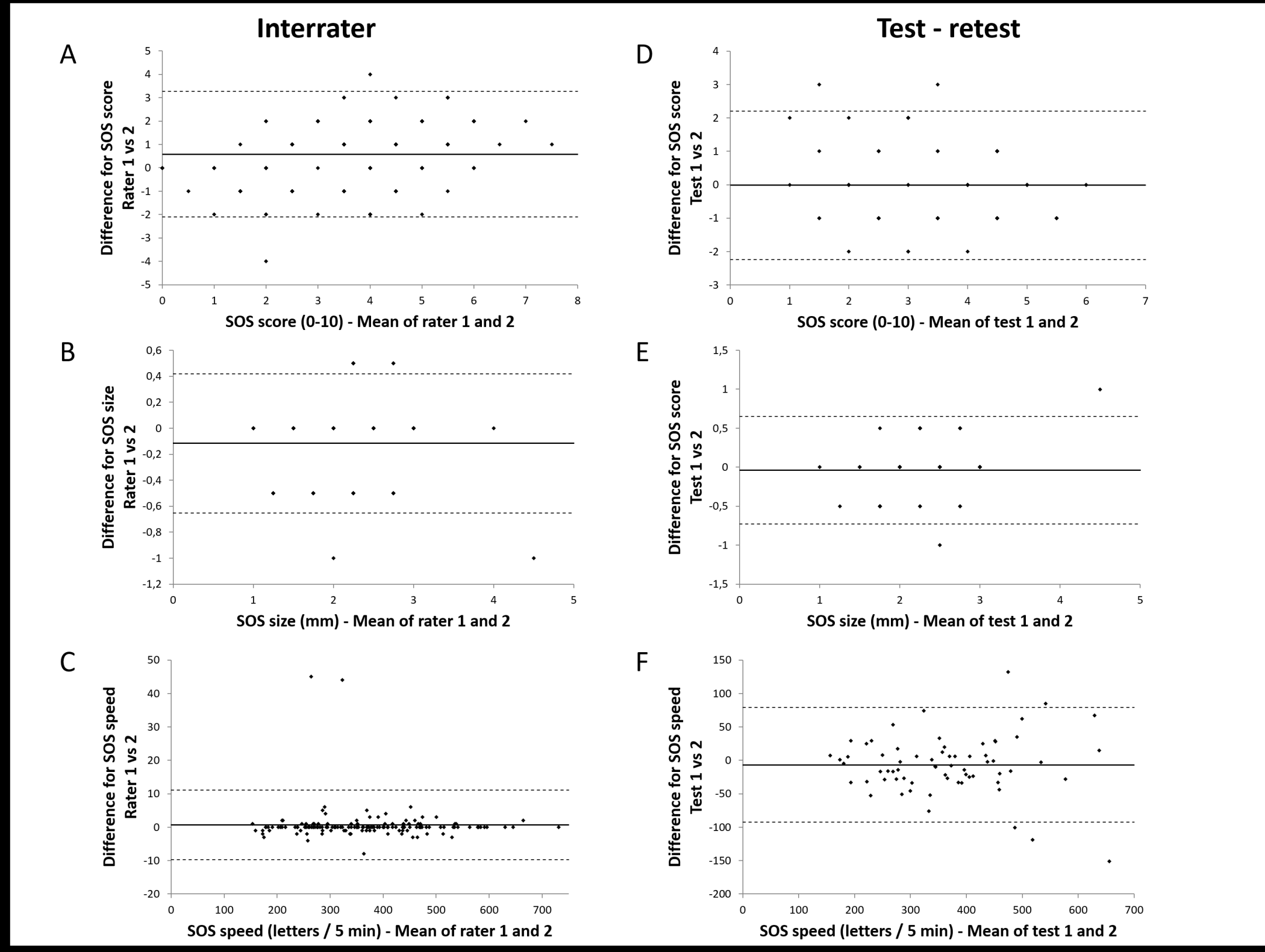

Supplement: S1 Fig — Panels A, B, D and E show less data points due to overlapping data. (TIF) [file pone.0173157.s002.tif]
