# Supplementary material for: Validity and reliability of a new tool to evaluate handwriting difficulties in Parkinson’s disease
Source: PLoS One. 2017 Mar 2;12(3):e0173157. doi: 10.1371/journal.pone.0173157 (PMC5333892; doi:10.1371/journal.pone.0173157)
Supplement: S1 Table — (DOCX) [file pone.0173157.s003.docx]

| Supplementary table 1: Reliability analysis of SOS-test main outcome variables | | | | | | | | |
| --- | --- | --- | --- | --- | --- | --- | --- | --- |
| Parameter SOS | **Scoring 1**  **Mean (SD)** | **Scoring 2**  **Mean (SD)** | **t value** | **p-value** | **Effect size** | **ICC (95% CI)** | **SEM** | **MDC** |
|  | **Intrarater reliability – rater 1** | | | | | | | |
|  | **PD** | | | | | | | |
| Speed | 367.7 ± 116.1 | 368.2 ± 116.0 | -0.828 | 0.409 | 0.005 | 0.999 (0.998 – 0.999) | 3.670 | 10.171 |
| Score | 3.5 ± 1.4 | 3.5 ± 1.4 | -0.304 | 0.762 | 0.014 | 0.915 (0.883 – 0.938) | 0.413 | 1.144 |
|  | **CT** | | | | | | | |
| Score | 2.5 ± 1.4 | 3.5 ± 2.1 | -3.759 | 0.001 | 0.558 | 0.769 (0.310 – 0.909) | 0.862 | 2.389 |
|  | **Intrarater reliability – rater 2** | | | | | | | |
|  | **PD** | | | | | | | |
| Speed | 368.3 ± 115.8 | 367.7 ± 116.1 | 1.881 | 0.062 | -0.005 | 1.000 (1.000 – 1.000) | 0.000 | 0.000 |
| Score | 4.1 ± 1.9 | 4.7 ± 1.8 | -6.117 | 0.000 | 0.330 | 0.848 (0.725 – 0.908) | 0.722 | 2.000 |
|  | **Scoring 1**  **Median (IQR)** | **Scoring 2**  **Median (IQR)** | **Wilcoxon z value** | **p-value** | **Effect size** | **Spearman correlation coefficient** |  |  |
|  | **Intrarater reliability – rater 1** | | | | | | | |
|  | **PD** | | | | | | | |
| Size | 2.5 (2.0 - 2.5) | 2.5 (2.0 - 2.5) | -2.191 | 0.028 | -0.124 | 0.855 |  |  |
|  | **CT** | | | | | | | |
| Speed | 492.0 (362.3 - 533.0) | 490.0 (363.0 - 533.0) | -0.170 | 0.865 | -0.024 | 0.997 |  |  |
| Size | 2.5 (2.5 - 3.0) | 2.5 (2.0-3.0) | -0.973 | 0.331 | -0.125 | 0.786 |  |  |
|  | **Intrarater reliability – rater 2** | | | | | | | |
|  | **PD** | | | | | | | |
| Size | 2.0 (2.0 - 2.5) | 2.0 (2.0 - 2.5) | -0.729 | 0.466 | -0.042 | 0.885 |  |  |
|  | **CT** | | | | | | | |
| Speed | 492.0 (363.0 - 533.0) | 492.0 (363.0 - 533.0) | -2.209 | 0.027 | -0.306 | 1.000 |  |  |
| Score | 3.5 (2.0 - 5.0) | 5.0 (3.5 - 6.0) | -3.241 | 0.001 | -0.449 | 0.856 |  |  |
| Size | 2.5 (2.0 - 3.0) | 2.5 (2.0 - 2.6) | -0.707 | 0.480 | -0.098 | 0.885 |  |  |
| Measurement units: SOS score = unit less (0-10); SOS speed = letters written in 5 minutes; SOS size = mm  Abbreviations: CT = healthy control; ICC = Intraclass Correlation Coefficient; IQR = interquartile range; SD = standard deviation; SEM = Standard Error of Measurement; MDC = Minimal Detectable Change; SOS = Systematic Screening of Handwriting Difficulties; PD = Parkinson’s disease; 95 % CI = 95 % confidence interval.  All ICC and Spearman correlation coefficients were significant at p < 0.001 | | | | | | | | |
